# Pouch excision, dysplasia and polypectomy in familial adenomatous polyposis ileal pouch anal anastomosis: a retrospective analysis

**DOI:** 10.1136/bmjgast-2025-001758

**Published:** 2025-12-09

**Authors:** Iain Blake, Hina Aslam, Shakil Ahmed, Muhammad Ahsan Javed, Paul Collins

**Affiliations:** 1 Department of Colorectal Surgery, Liverpool University Hospitals NHS Foundation Trust, Liverpool, England, UK; 2 Institute of Systems, Molecular and Integrative Biology, University of Liverpool, Liverpool, UK; 3 Department of Gastroenterology, Liverpool University Hospitals NHS Foundation Trust, Liverpool, UK; 4 North West Endoscopy training academy, Liverpool John Moores University, Liverpool, UK

**Keywords:** FAMILIAL ADENOMATOUS POLYPOSIS, DYSPLASIA, ILEOANAL POUCH, POLYP, ENDOSCOPIC POLYPECTOMY

## Abstract

**Introduction:**

Familial adenomatous polyposis (FAP) is a rare autosomal dominant syndrome that, if untreated, carries a 100% lifetime risk of developing colorectal cancer. Proctocolectomy with ileal pouch–anal anastomosis (IPAA) is a surgical approach for FAP management, but data on long-term outcomes remain limited. This study aimed to assess IPAA-associated pouch excision, dysplasia, polyp management and pouchitis in patients with FAP post IPAA.

**Methods:**

Single-centre retrospective review of patients with FAP with IPAA at a tertiary referral centre. Data on demographics, pouch excision, endoscopic and histopathological records were collected. Statistical analysis was performed using Excel and GraphPad.

**Results:**

Forty patients with FAP with IPAA were included. The median age was 48 years (IQR 33–58.5), with a median age of 22 years at pouch formation (IQR 18–30.5) and a median pouch age of 217 months (IQR 143–279). A total of 230 lower gastrointestinal endoscopies were assessed. Low-grade dysplasia was identified in 70 endoscopies, and polyps in 82, with the pouch being the most common site. One case of rectal adenocarcinoma was identified, with no cases of high-grade dysplasia. There were 35 episodes involving polypectomy, with cold snare being the most common method (50%). Endoscopic management had a lower complication rate than surgical management (p=0.0474). Eight (20%) patients underwent pouch excision, with poor function being as common as pouch-related complications (37.5%), with a median pouch age at excision of 167 months (IQR 115.5–286.5). Endoscopic pouchitis was significantly more common in those who underwent pouch excision (p=0.0231).

**Conclusion:**

Patients with FAP and IPAA require surveillance due to a high incidence of dysplasia and polyp formation, although malignancy remains rare. There is a significant rate of pouch excision, for which pouchitis appears to be a risk factor. Early and aggressive endoscopic management of polyps is recommended to prevent advanced disease and minimise complications associated with surgical approaches.

**WHAT IS ALREADY KNOWN ON THIS TOPIC**

- Patients with familial adenomatous polyposis (FAP) with ileal pouch anal anastomosis (IPAA) develop polyps and dysplasia and require surveillance due to the ongoing risk of adenocarcinoma. There are limited data regarding polyp management and reasons for pouch loss in patients with FAP.

**WHAT THIS STUDY ADDS**

- This study provides a long-term follow-up for a rare cohort of patients. It identifies that rates of pouch excision are often not related to polyp-associated pathology, and that pouchitis is a significant risk factor for pouch excision.

**HOW MIGHT THIS STUDY AFFECT RESEARCH, PRACTICE OR POLICY**

- This study emphasises the need for specialist follow-up of FAP-related IPAA, particularly with respect to early endoscopic management of polyps.

## Introduction

 Familial adenomatous polyposis (FAP) is an autosomal dominant condition characterised by the development of numerous gastrointestinal (GI) adenomatous polyps alongside distinct extraintestinal manifestations.^1^ If left untreated, FAP confers a lifetime risk of colorectal cancer of 100%. FAP is a rare condition, accounting for less than 1% of all colorectal cancers, with an estimated incidence of 1 per 8300 births.^2^

FAP arises from mutations in the tumour-suppressing adenomatous polyposis coli (APC) gene, located on chromosome 5q.^3 4^ APC encodes a 312 kDa multifunctional protein crucial for the degradation of β-catenin, thereby negatively regulating the Wnt signalling pathway involved in cell proliferation, apoptosis and tissue homeostasis.^3^ Beyond Wnt signalling, APC plays roles in actin and microtubule dynamics and chromosomal segregation during mitosis.^3 5^

APC mutation results in β-catenin accumulation and dysregulated Wnt pathway activation^3^, initiating the classical adenoma-to-carcinoma progression characteristic of colorectal cancer. Additionally, errors accumulated during cell division through its role in chromosomal segregation and microtubule dynamics contribute to genetic instability.^5^ Together, these mechanisms underlie the development of hundreds of adenomatous GI polyps and, eventually, colorectal adenocarcinoma. Most individuals with FAP have developed colorectal cancer by the fourth or fifth decade of life.^1^

Prophylactic surgery is a cornerstone of FAP management. The three principal surgical options include proctocolectomy with ileal pouch anal anastomosis (IPAA), colectomy with ileorectal anastomosis and panproctocolectomy with end ileostomy.^2^ The original IPAA technique, first described by Parks and Nicholls in 1978,^6^ involved a mucosectomy with hand-sewn ileoanal anastomosis.^7^ Advances, including the introduction of the J-pouch by Utsunomiya *et al* in 1980^8^ and the widespread adoption of stapled anastomosis, have established the stapled J-pouch technique as the current standard for IPAA.^7^

Despite surgical intervention, the risk of retained rectal tissue and high penetrance of APC mutations necessitate ongoing surveillance as the risk of malignancy remains.^9^ Given the rarity of FAP and the prolonged follow-up intervals required, data on long-term outcomes, surveillance protocols, polyp management and pouch-specific complications remain limited. This study aimed to evaluate rates of pouch excision, dysplasia, polyp occurrence and management in patients with FAP who underwent IPAA.

## Methods

### Study population

Patients with FAP were identified from an existing departmental database of patients with FAP at Liverpool University Hospitals NHS Foundation Trust and their records reviewed to determine if they had undergone IPAA. In addition, the records of patients who had attended the nurse-led pouch service were scrutinised to identify those with FAP and IPAA.

Patients were included if they had either undergone IPAA since 2002 or been followed up since 2002 by the FAP/pouch service in cases of IPAA performed prior to this date. A retrospective, manual chart review of electronic clinical records, histopathology and endoscopy reports was conducted. No natural language processing was used. This study was reported in accordance with the Strengthening the Reporting of Observational Studies in Epidemiology (STROBE) guidelines (checklist available in online supplemental materials).^10^

### Data collection

The following data points were extracted:

Patient demographics: sex, age, date of pouch formation and cause/date of death.

Pouch-related outcomes: reason and date of pouch excision, number of pouch endoscopies performed and identification of dysplasia (low-grade or high-grade), carcinoma, endoscopic pouchitis or polyps.

Polyp-related data: polyp burden identified at time of endoscopy, techniques used for polyp removal and complications following polypectomy.

### Analysis

All data were collated onto an Excel spreadsheet for analysis, with GraphPad used for statistical testing. Fisher’s exact test was used to assess categorical variables. A p value of <0.05 was considered statistically significant.

The records for each patient were categorised as either having full or partial endoscopic follow-up. Every endoscopy performed at our facility for each patient was included in this study. Patients were deemed to have full endoscopic follow-up if all endoscopic records were available and endoscopy was documented at regular intervals (mean time between lower GI endoscopy <36 months) from the time of pouch formation or the earliest available electronic records (circa 2002).

Patients were deemed to have partial endoscopic follow-up if they had fewer endoscopies available, either due to missed procedures or procedures performed at other facilities, which could not be independently verified.

## Results

### Patient demographics

A total of 41 patients with FAP who underwent IPAA were identified, with one excluded due to an absence of clinical, endoscopic or histological data. Among the remaining 40 patients, 21 (52.5%) were female and 19 (47.5%) were male. At the time of analysis, five patients were deceased.

The median age of the cohort (at the time of analysis or death) was 48 years (IQR 33–58.5). The median age at the time of pouch formation was 22 years (IQR 18–30.5). The median pouch age for all patients was 217 months (IQR 143–279).

### Mortality and pouch loss

Five patients were deceased at the time of analysis, with a median age at death of 52 years (IQR 42.5–58.5). Causes of death are summarised in table 1. One (2.5%) patient developed rectal adenocarcinoma (T3N0M0) and died from progressive disease. The pouch age at the time of cancer diagnosis was 341 months. This patient had undergone IPAA at another centre and had been lost to follow-up, with no recent endoscopic or clinical data available for review.

**Table 1:** Causes of death in patients with FAP with IPAA and age at time of death

Mortality (n=5)	Cause of death	Age at death (years)
	High-grade neuroendocrine tumour	52
	Cancer of unknown primary (likely small bowel)	59
	Jejunal adenocarcinoma	58
	Rectal adenocarcinoma	49
	Unknown	36

FAP, familial adenomatous polyposis; IPAA, ileal pouch anal anastomosis.

Eight patients underwent pouch excision (20%), and one additional patient had their pouch defunctioned with an ileostomy secondary to small bowel perforation. The median age of the pouch at the time of excision or defunctioning was 165 months (range: 41–345 months). Most procedures were performed electively (66.7%).

Pouch excision for poor function (n=3, 37.5%) was as common as pouch-related complications (n=3, 37.5%). A summary of causes is provided in table 2.

**Table 2:** Causes of IPAA loss or defunctioning in FAP, elective versus emergency treatment and pouch age at time of procedure

IPAA excision (n=8)	Reason for procedure	Elective vs emergency	Pouch age (months) at procedure
	Poor function	Elective	41
	Poor function	Elective	165
	Poor function	Elective	169
	Fistula	Elective	138
	Obstructing stricture and bleeding	Elective	267
	Low rectal adenocarcinoma	Elective	345
	Unknown	Unknown	48
	Small bowel obstruction with necrotic pouchitis	Emergency	402
IPAA defunctioning (n=1)	Spontaneous small bowel perforation	Emergency	84

FAP, familial adenomatous polyposis; IPAA, ileal pouch anal anastomosis.

### Dysplasia, polyps and pouchitis

Of the 40 patients in the study, full endoscopic follow-up data were available for 22, while 14 had partial endoscopic follow-up. No endoscopy reports were accessible for four patients. A total of 230 lower GI pouch endoscopies were included in the analysis. The total mean number of endoscopies per patient was 6.4 endoscopies per patient (8.2 for those with full follow-up data, 3.6 for those with partial follow-up data). Low-grade dysplasia (LGD) was detected on 70 occasions, corresponding to an incidence rate of one detection per 3.29 endoscopies. A total of 27 (67.5%) patients were identified as having LGD. Of patients who had IPAA performed post 2002 for which full endoscopic data were available (n=18), 66.7% developed LGD with a median time to development of 108 months (range 20–183 months). Notably, no cases of high-grade dysplasia (HGD) were identified. The pouch was the most common site for detecting LGD, followed by the rectal cuff. Dysplasia was identified via polypectomy, routine biopsy and pouch excision, with polypectomy being the most frequent method (52.2%).

**Figure 1:**
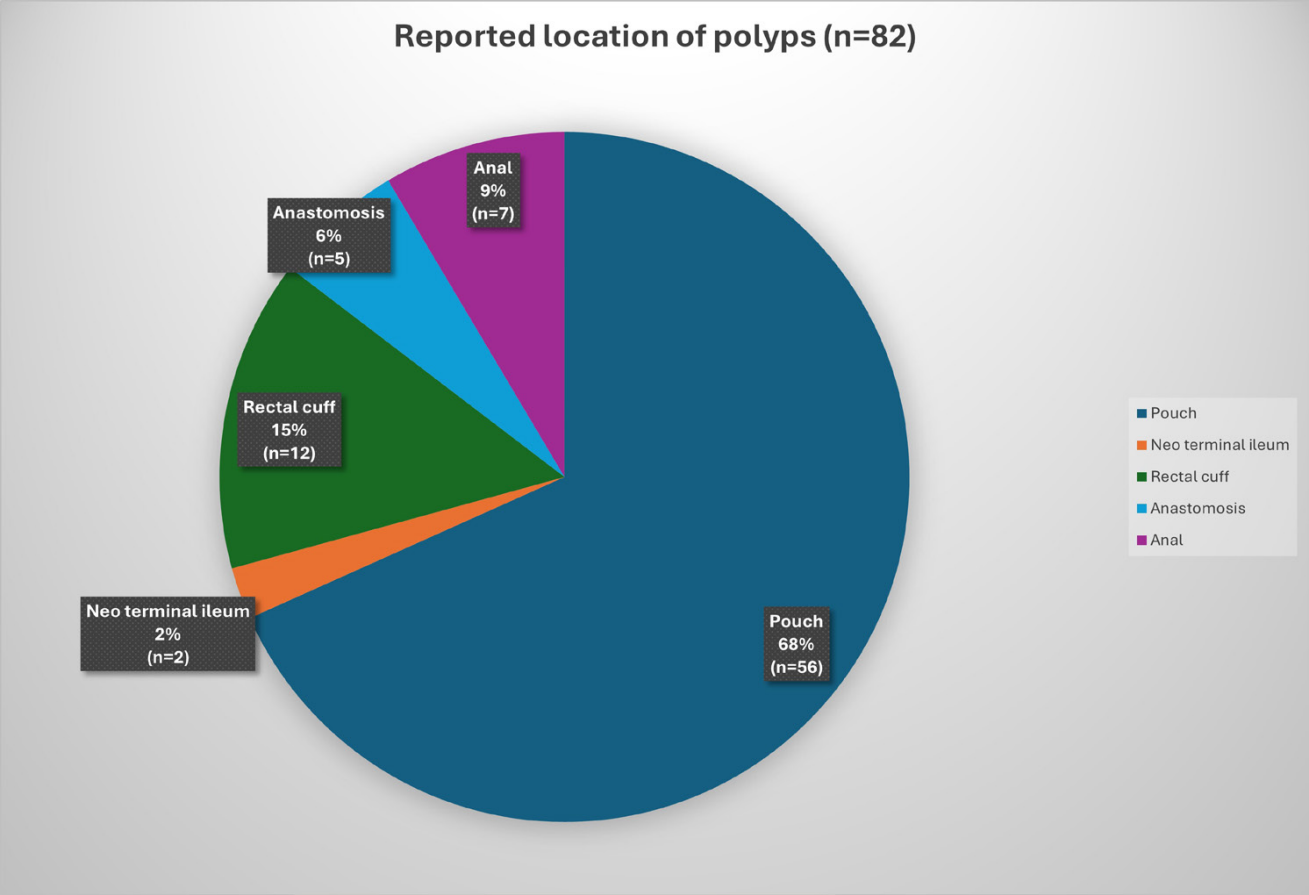
The reported location of polyps in patients with familial adenomatous polyposis with ileal pouch–anal anastomosis. Data from 82 lower gastrointestinal endoscopies.

Polyps were observed in 82 endoscopies, equating to a polyp detection rate of one per 2.8 endoscopies. However, accurate assessment of polyp burden was limited due to variability in reporting terminology, with descriptions ranging from quantitative to qualitative. A total of 27 (67.5%) patients were identified as having polyps, of which 25 (92.6%) were adenomas. Of patients who had IPAA performed post 2002 for which full endoscopic data were available (n=18), 66.7% developed polyps with a median time of 90 months (range 3–212 months). The pouch was the most common site for polyps, followed by the rectal cuff (figure 1).

There were 35 recorded episodes of polypectomy, defined as the removal of one or more polyps during an endoscopy. Patients who did not undergo immediate polypectomy were followed up. Significant variability in terminology was observed in documenting the number and size of polyps removed, precluding the ability to standardise findings regarding the total number of polyps removed during each procedure. Among the 35 polypectomy episodes, five methodologies (cold snare, cold biopsy, hot snare, transanal and endoscopic mucosal resection (EMR)) were documented a total of 36 times, reflecting procedures in which more than one technique was employed.

**Figure 2:**
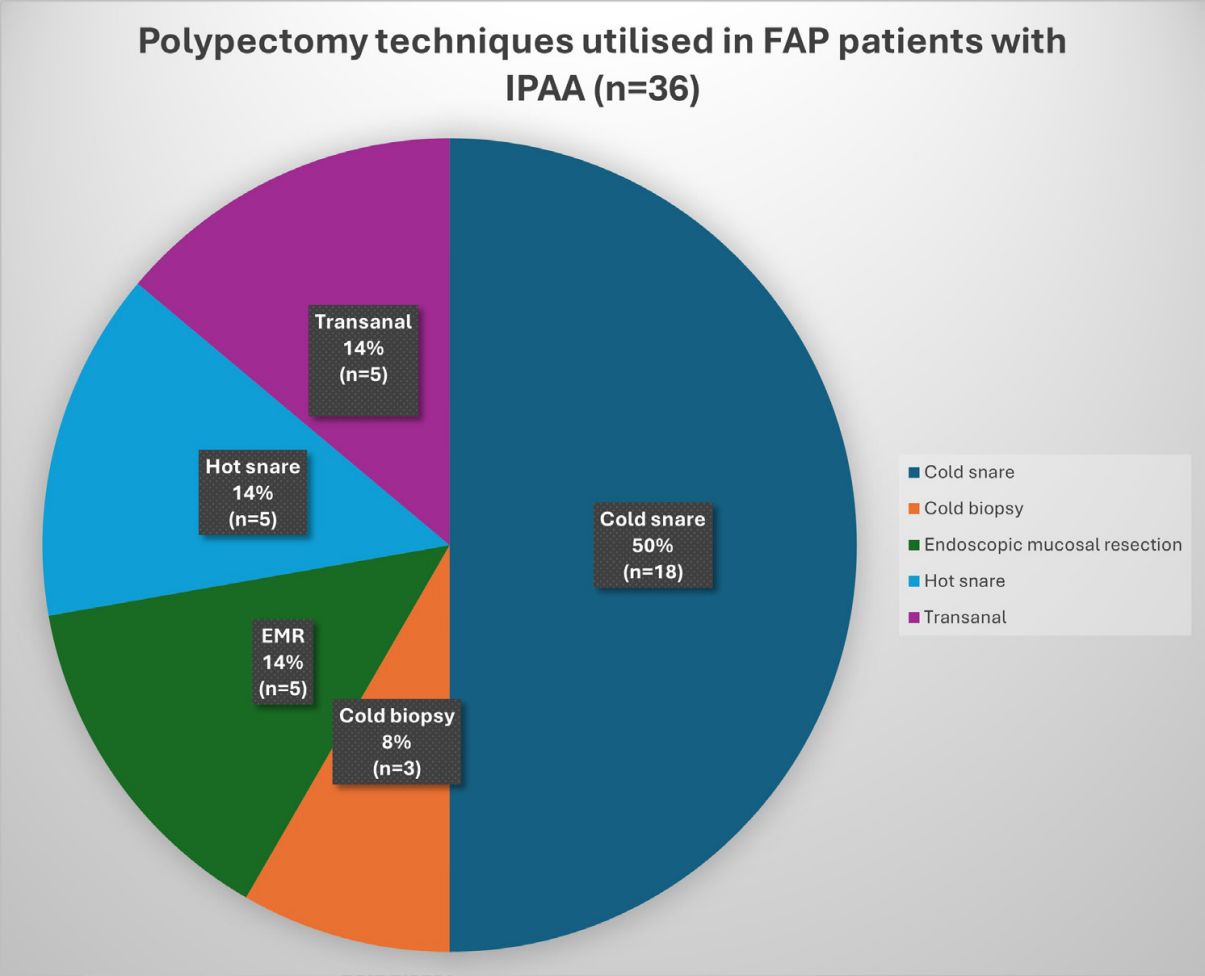
Percentage distribution of techniques used for polypectomy in patient with FAP with IPAA (data from 35 polypectomy episodes). FAP, familial adenomatous polyposis; IPAA, ileal pouch anal anastomosis.

Cold snare polypectomy was the most used technique, accounting for 50% of cases (figure 2). Three postpolypectomy complications were reported: one episode of self-limiting postpolypectomy bleeding following EMR, representing 20% of EMR cases, and two perforations during transanal surgical polypectomy under anaesthesia, accounting for 40% of transanal cases. Endoscopic management had a significantly lower complication rate than transanal management (3.22% vs 40%, p=0.0474 Fisher’s exact test two tailed).

Among patients with full or partial endoscopic follow-up (n=36), 11 were diagnosed with endoscopic pouchitis (30.6%). Endoscopic pouchitis was significantly more common in patients who underwent pouch excision compared with those whose pouches were retained (p=0.0231, Fisher’s exact test, two tailed). All patients who had pouch excision for poor function showed endoscopic and histological evidence of pouchitis.

## Discussion

Our study found no cases of HGD and only one instance of rectal adenocarcinoma, likely due to retained rectal tissue at the cuff following pouch formation. However, we observed significant rates of LGD and polyps, consistent with findings from published studies.^10^,^12^ A recent systematic review^11^ reported pouch adenoma rates ranging from 5.9% to 53.2%, with cumulative risks of 12% and 58% at 5 and 20 years. In addition, they identified a total of 45 IPAA-related cancers since 1994, 15 of which occurred in the rectal cuff or anastomosis.^11^ Our case of rectal adenocarcinoma contributes to this small cohort.

Patel and colleagues’^12^ recent study of 249 patients reported that 76% developed adenomas, with 18% developing advanced cuff lesions. Their statistical analysis indicated a higher risk of advanced lesions in patients with older pouches and with longer surveillance intervals. Similarly, a retrospective study by Tajika and colleagues^13^ identified LGD in 21 out of 24 patients with FAP-IPAA, with incidence increasing with age. This aligns with Moussata *et al*’s^14^ earlier findings, showing 78% of patients with FAP-IPAA developed polyps, 70% of which were LGD, and 4% were HGD. A separate study estimated a 10-year carcinoma risk of 1%.^15^ Our findings reflect these trends, with no cases of HGD observed and a single case of rectal adenocarcinoma occurring in a patient who had not undergone regular endoscopic surveillance.

Our results underline that while LGD and polyps are common post-IPAA in patients with FAP, HGD and malignancy remain rare. The findings highlight the importance of regular surveillance and aggressive polyp management, particularly around the anal transition zone due to the risk of retained rectal tissue, to prevent progression to advanced lesions and malignancy.

### Surveillance and compliance challenges

Current UK guidelines recommend pouchoscopy every 1–3 years.^16^ However, challenges to regular surveillance persist. Lifelong FAP management may involve patient movement between centres, potentially disrupting follow-up. Additionally, data on patient compliance with surveillance are limited. One study^17^ reported poor surveillance compliance in 20% of patients with FAP postsurgery, while another study^18^ noted 8% self-reported non-compliance in patients with FAP-IPAA. It is unclear whether our patients with missing endoscopy data were due to poor compliance or procedures being performed in other centres. These findings emphasise the need for robust systems to ensure regular monitoring. Managing patients with FAP in specialised tertiary centres, as recommended by the Rare Disease Collaborative Network^19^, may improve consistency and outcomes.

### Pouch excision and pouchitis

A subset of our patients underwent pouch excision, primarily for non-polyp-related pathology. Aelvoet *et al*^20^ reported an 8% pouch excision rate for polyposis or malignancy in patients with IPAA-FAP with a median time to excision of 305 months. Our data indicate that while pouch excision due to polyp-related pathology does occur, a significant proportion is due to functional issues or non-polyp-related abdominal problems. This may explain our shorter median time to IPAA excision of 167 months.

Notably, endoscopic pouchitis emerged as a significant risk factor for pouch excision. Pouchitis is known to impair pouch function.^21^ FAP-related IPAA is traditionally thought to have low rates of pouchitis, with a 2021 meta-analysis showing a pooled prevalence rate of 6% compared with 32% in ulcerative colitis.^22^ This lower risk has been confirmed in a further systematic review.^23^ Our data showed that FAP-related IPAAs may have a high rate of pouchitis, and this is a potentially significant and modifiable risk factor for excision. We did not evaluate non-operative management of pouchitis in this study; further work is needed to address this. Given our limited sample size and single-centre nature, further research on a larger cohort is needed to validate these findings. Effective long-term management of recurrent pouchitis remains challenging, underscoring the need for consistent follow-up and tailored treatment strategies within specialised centres.

### Polyp management techniques

The presence of diminutive polyps is expected in patients with FAP. Although the evidence base is limited, it is generally accepted that routine biopsy or polypectomy of all FAP-associated polyps is not standard practice. The European FAP Consortium surveillance strategy supports selective polypectomy, specifically for lesions >5 mm in the prepouch ileum or pouch body, >3 mm in the rectal cuff, or for those with high-risk features suggestive of HGD, with follow-up endoscopy at lengths of up to 2 years.^24^ This may account for why polyps were observed in more endoscopies than those in which polypectomy occurred. While we recognise that observing diminutive polyps carries risk, the lack of HGD identified supports this strategy.

Various techniques were employed for polyp management, with cold snare polypectomy being the most common. Data on polypectomy techniques in patients with FAP-IPAA are sparse. A 2022 retrospective study by Tajika *et al*^25^ reported a median of five polypectomies per patient over a mean follow-up of 11.3 years. Unlike our study, where cold snare was the predominant method, they found 96.7% of cases used argon plasma coagulation, with significantly lower rates of cold polypectomy and a 10% postpolypectomy bleeding rate. Differences in techniques are likely to reflect advancements in endoscopic technology and practices over the surveyed timeframes.

Our data show polypectomy in FAP-IPAA is generally safe, with low complication rates for endoscopic techniques. Transanal excision was reserved for distal pouch polyps deemed unresectable endoscopically, though this approach does carry an increased risk of serious complications, such as perforation. These findings underscore the necessity of regular endoscopy and management of polyps effectively and early, with an emphasis on minimally invasive advanced endoscopic techniques.

### Strengths and limitations

This study has notable strengths, including a well-defined population of patients with FAP with IPAA over an extended timeline and a detailed review of clinical, endoscopic and histological data. However, limitations include a relatively small sample size, incomplete data for some patients and the retrospective single-centre design that may lead to the introduction of bias. Additionally, variability in terminology for dysplasia and polyp assessment hindered standardised data interpretation. To address this, we strongly advocate for the adoption of standardised endoscopic reporting criteria and adherence to management strategies, such as the strategy proposed by the European FAP Consortium.^24^ Furthermore, given this rare patient cohort, future work should prioritise a multicentre study with endoscopy tracking to gain further insight into the long-term outcomes and complications associated with IPAA.

## Conclusion

Our study adds to the limited body of evidence on outcomes in patients with FAP with IPAA. While LGD and polyps are common, malignancy and HGD remain rare. Based on our findings, we recommend the following for managing FAP-related patients with IPAA :

1. Adoption of standardised endoscopic reporting criteria to enhance consistency and accuracy of surveillance data.
2. Management by experienced pouch endoscopists within specialised tertiary centres, adhering to European FAP Consortium guidelines.
3. Further research to assess the impact of pouchitis on FAP-related IPAA excision rates, in conjunction with management strategies to minimise its impact on pouch function and patient outcomes.

Ultimately, consistent surveillance and specialised management of patients with FAP-IPAA is critical to optimising long-term outcomes.

## Supplementary material

10.1136/bmjgast-2025-001758 online supplemental file 1

## Data Availability

No data are available.
